# Occurrence and Estimation of *trans*-Resveratrol in One-Year-Old Canes from Seven Major Chinese Grape Producing Regions 

**DOI:** 10.3390/molecules16042846

**Published:** 2011-03-31

**Authors:** Ang Zhang, Yulin Fang, Xuan Li, Jiangfei Meng, Hua Wang, Hua Li, Zhenwen Zhang, Zhijun Guo

**Affiliations:** 1 College of Enology, Northwest A&F University, Yangling, Shaanxi 712100, China; 2 Shaanxi Engineering Research Center for Viti-Viniculture, Yangling, Shaanxi 712100, China; 3 Department of Biological and Agricultural Engineering, University of California, Davis, One Shields Avenue, CA 95616, USA

**Keywords:** *trans*-resveratrol, grape, one-year-old canes, HPLC

## Abstract

The concentration of *trans*-resveratrol in 165 grape cane samples from three major grape production regions and four large distribution centers of Chinese wild *Vitis* species were determined by reversed-phase high-performance liquid chromatography (HPLC). Among the different genotype groups and purpose of uses, cultivars of *V. vinifera* had much higher amounts of *trans*-resveratrol than did the cultivars of both *V. labrusca* or *V. labrusca* and *V. vinifera* hybrids, and within the *V. vinifera* species, significantly higher amounts of *trans*-resveratrol were found in wine grapes compared to table ones. No significant differences were observed between *V. labrusca* and its hybrids from crosses with *V. vinifera*, and between red cultivars and white ones (*P* < 0.05 or *P* < 0.01). The contents of *trans*-resveratrol, as a normal constituent occurring in grape canes, in Chinese wild species of *V. amurensis*, *V. pentagona*, and *V. davidii* from their native habitats were also relatively high.

## 1. Introduction

*trans*-Resveratrol (3,5,4'-trihydroxystilbene) is a phytoalexin synthesized naturally by several plants in response to pathogen infection, traumatic damage, ultraviolet (UV) irradiation, and other stresses [1,2,3]. Its occurrence has been documented in a narrow range of dietary sources of which grapes, peanuts, blueberries, strawberries, hops, and their products are the main representatives [4,5,6,7,8,9]. Moreover, *trans*-resveratrol has also been produced by chemical synthesis and is sold as a nutritional supplement derived primarily from the Chinese and Japanese folk medicine giant knotweed rhizome [10]. It has gained significant global attention due to its promising biological and pharmacological properties including antioxidant, anticancer, cardioprotective, anti-inflammatory, antiplatelet, antiviral action and life span extension in diverse organisms, from yeast to vertebrates [4,11,12,13,14,15,16,17].

**Figure 1:**
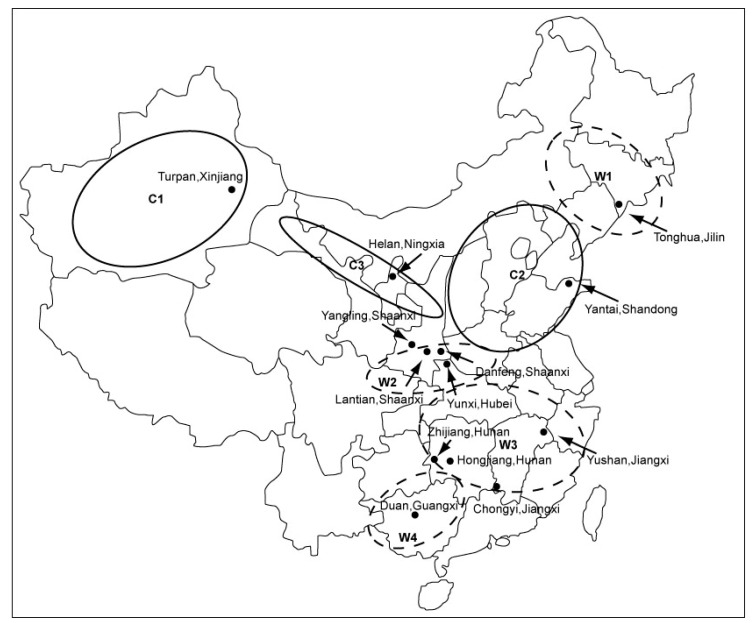
Geographical location of the sampling sites (black points).

Grapes and wine are the primary dietary sources of resveratrol in the human diet. In the past 20 years, China achieved much success in the development of its grape and wine industry. All the cultivated grape cultivars in China are mainly grown in three major regions, indicated as C1, C2, and C3 (Figure 1). Most Chinese wild *Vitis* species are distributed in four major eco-geographic centers, labeled W1, W2, W3, and W4 (Figure 1).

Provinces with a sizeable amount of grape cultivation in China are Xinjiang, Hebei, Shandong, Liaoning and Henan [18]. Additionally, China is one of the most important centers of origin of *Vitis* species and several wild Chinese *Vitis* species that occur widely in their native habitats, such as *V. amurensis* in the north-east regions of China, *V. davidii* and *V. pentagona* in the southern regions, have been developed to produce wine [19,20]. Large quantities of grape canes are produced as solid wastes during the annual pruning campaigns of the viticulture industry. Compared with other solid by-products from wineries and vineyards such as grape pomace [21], grape stems [22], grape seeds [23], and so on, people have paid little attention to grape cane wastes with respect to the possible recovery of high value-added compounds. These pruning wastes represent a great potential source of natural antioxidants, and after suitable treatment these low-cost residues could contribute to the sustainable development of related industries. Currently, these grape cane wastes are disposed of in landfills, burned *in situ* or used as fuels, indicating low-value utilizations [24]. We know of only a few previous studies that have addressed the resveratrol content in a single sample of Pinot Noir (*V. vinifera*) grape cane [25,26]. The diversity of the health-promoting properties of *trans*-resveratrol has sparked intense research interest devoted to developing resveratrol-enriched foodstuffs or plants and exploiting other new potential sources to meet the increasing demand [27,28,29].

The main objectives of the present work were: (i) to investigate and evaluate the *trans*-resveratrol content in one-year-old canes at the grape germplasm level in order to acquire information for the future utilization of these wastes; (ii) to compare grape canes with other main known sources of *trans*-resveratrol; (iii) to estimate the annual yield and the potential economic value of *trans*-resveratrol in grape cane wastes from seven large grape-concentrating areas of China.

## 2. Results and Discussion

### 2.1. Genotypic variation of trans-resveratrol contents in grape canes from two germplasm repertoires

**Figure 2:**
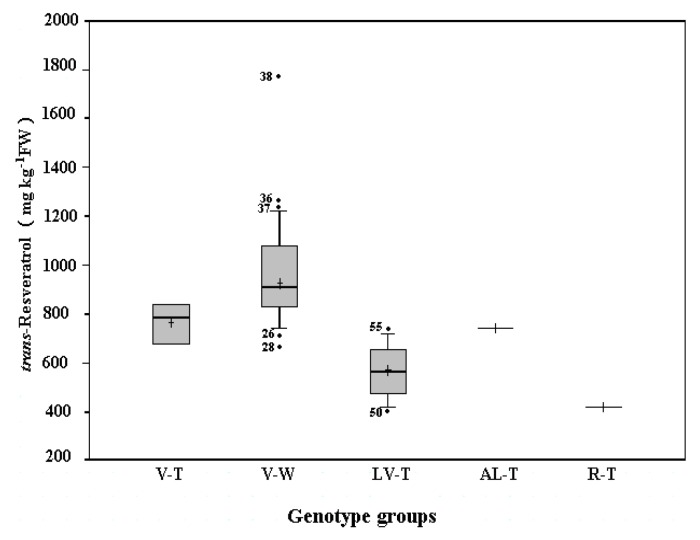
Box and whisker plots of Yangling Grape Germplasm Repository. The median and mean value are indicated by the horizontal bar and the plus sign inside the box, respectively; the height of the box represents the interquartile range (IQR), which is the difference between the third quartile and the first quartile of the data. The whisker (the vertical lines from the top and bottom of the box) extends to a distance of 1.5 times IQR. Data outside these whiskers are marked by the black dots. The numbers in front of the black dots represent the accession numbers (Table 1). V-T, table grape cultivars of *V. vinifera*; V-W, wine grape cultivars of *V. vinifera*; LV-T, table grape cultivars of hybrids between *V. labrusca* and *V. vinifera*; AL-T, table grape of hybrid between *V. aestivalis* and *V. labrusca*; R-T, table grape of *V. rotundifolia*.

**Figure 3:**
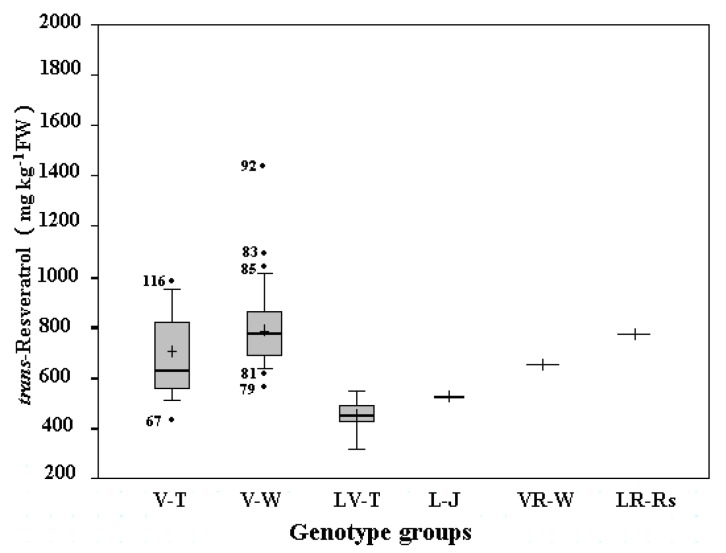
Box and whisker plots of Yantai Grape Germplasm Repository. The median and mean value are indicated by the horizontal bar and the plus sign inside the box, respectively. The height of the box represents the interquartile range (IQR), which is the difference between the third quartile and the first quartile of the data. The whisker (the vertical lines from the top and bottom of the box) extends to a distance of 1.5 times IQR. Data outside these whiskers are marked by the black dots. The numbers in front of the black dots represent the accession numbers (Table 1). V-T, table grape cultivars of *V. vinifera*; V-W, wine grape cultivars of *V. vinifera*; LV-T, table grape cultivars of hybrids between *V. labrusca* and *V. vinifera*; L-J, juice grape cultivars of *V. labrusca*; VR-W, wine grape cultivar of hybrid between *V.vinifera* and *V. riparia.* LR-Rs, rootstock grape cultivar of hybrid between *V. labrusca* and *V. riparia*.

A total of 118 grape cultivars (51 from Yangling and 67 from Yantai), mainly belonging to three important commercial grape groups: *V. vinifera*, *V. labrusca*, and *V. labrusca* and *V. vinifera* hybrids, were analyzed for *trans*-resveratrol content. The frequency distribution and median of amounts of *trans*-resveratrol in one-year-old canes were similar in the two repositories (Figure 2 and Figure 3).

The wine grape cultivars of *V. vinifera* had higher *trans*-resveratrol contents than other grape genotypic groups in this study in the two repositories. *trans*-Resveratrol content in wine grape cultivars of *V. vinifera* ranged from 664.7 to 1,751.6 mg kg^−1^ of cane fresh weight (FW) with a median of 906.6 mg kg^−1^ of cane FW in Yangling (Figure 2) and from 570.8 to 1,452.9 mg kg^−1^ of cane FW with a median of 780.9 mg kg^−1^ of cane FW in Yantai (Figure 3). The *trans*-resveratrol concentrations posted by wine grape cultivars of *V. vinifera* varied significantly with their genetic backgrounds. The highest value was found in ‘Pinot Noir’, one of the most famous wine grape cultivars, reaching 1,751.6 and 1,452.9 mg kg^−1^ of cane FW in Yangling and Yantai, respectively. It is somewhat similar to the previous reports that the highest *trans*-resveratrol concentrations were also found in red wines from cv. ‘Pinot Noir’ [30,31].

Other wine grapes also produced quite high values, such as ‘Cabernet Gernischt’ and ‘Cabernet Sauvignon’ from Yangling and ‘Riesling’ and ‘Silvaner’ from Yantai, reaching 1,273.4 and 1,238.6 mg kg^−1^ of cane FW and 1,072.2 and 1,027.4 mg kg^−1^ of cane FW, respectively. ‘Müller-Thurgau’ from Yangling and ‘Jiubai’ from Yantai were found to have the lowest values of 664.7 and 570.8 mg kg^−1^ of cane FW, respectively.

**Table 1:** Locations and characteristics of grape cultivars used in this study.

	Locations	Species^a^		Color	Usage	No.	Cultivars or Genotypes^c^
	Turpan	V		Green	Raisin	3	Centennial Seedless (1), Manaizi (2), Thompson Seedless (3)
				Red	Raisin	4	Crimson Seedless (4), Monukka (5), Ruby Seedless (6), Munage (7)
	Helan	V		Green	Wine	1	Chardonnay (8)
				Red	Wine	7	Cabernet Franc (9), Cabernet Gernischt (10), Cabernet Sauvignon (11), Merlot (12), Syrah (13), Pinot Noir (14), Gamay (15)
	Yangling	V		Green	Table	2	Pearl of Csaba (16), Queen of the Vineyard (17)
					Wine	15	Aligoté (18), Angelina (19), Augusta (20), Baibigebuer (21), Baidehai (22), Bourboulenc (23), Chardonnay (24), Ecoly (25), Gouais Blanc (26), Itlian Riesling (27), Müller-Thurgau (28), Petit Manseng (29), Pollux (30), Sauvignon Blanc (31), Semillon (32)
				Red	Wine	16	8804 (33), Blue French (34), Cabernet Franc (35), Cabernet Gernischt (36), Cabernet Sauvignon (37), Pinot Noir (38), Syrah (39), Gamay (40), Merlot (41), Granoir (42), Gewürztraminer (43), Muscat Hamburg (44), Carignane (45), Cinsaut (46), Zinfandel (47), Roussanne Du Var (48)
					Table	1	Shandongzaohong (49)
		LV		Green	Table	2	Golden Queen (50), Hakuho (51)
				Red	Table	13	Beni Zuiho (52), Hutai8 (53), Ikawa1014 (54), Ikawa1025 (55), Iona (56), Izunishiki (57), Kyoho (58), Campbell Early (59), Alirobar (60), Beni Fuji (61), Honey Red (62), Jasmine (63), Tensyu (64)
		AL		Red	Table	1	Conquistador (65)
		R		Red	Table	1	Alachua (66)
	Yantai	V		Green	Table	7	Victoria Blanc (67), Autumn White (68), Xiabai (69), Zaobai (70), Jingyu (71), Niunai (72), Zexiang (73)
					Wine	13	Chardonnay (74), Chenin Blanc (75), Colombard (76), Gamay Blanc (77), Grenache Blanc (78), Jiubai (79), Muscat Blanc (80), Muscat of Alexandria (81), Pinot Blanc (82), Riesling (83), Rkatsiteli (84), Silvaner (85), Ugni Blanc (86)
				Red	Wine	20	Cabernet Franc (87), Cabernet Gernischt (88), Cabernet Sauvignon (89), Gamay (90), Merlot (91), Pinot Noir (92), Syrah (93), Nebbiolo (94), Petit Verdot (95), Pinot Gris (96), Mission (97), Ruby Cabernet (98), Sangiovese (99), Saperavi (100), Noir de Maisky (101), Flame Muscat (102), Alicante Bouschet (103), Yan73 (104), Yan74 (105), Jasmin (106)
					Table	14	Autumn Black (107), Autumn Royal (108), Black Rose (109), Heijixin (110), Jingxiu (111), Guibao (112), Lungyen (113), Manicure Finger (114), Muscat Mathiasz Janosne (115), Fenghuang 51 (116), Hongxiangjiao (117), Rizamat (118), Red Globe (119), Red Guibao (120)
		LV	Green		Table	1	Triumph (121)
			Red		Table	8	Fujiminori (122), Jingya (123), Jingyou (124), Meiguilu (125), Olympia Black (126), Takasumi (127), Wase Takasumi (128), Fox (129)
		L	Green		Juice	1	Moore's Diamond (130)
			Red		Juice	1	Concord (131)
		VR	Red		Wine	1	Bacco Noir (132)
		LR	Red		Rs^b^	1	Beta (133)
Lantian		P	Red		Wine^§^	3	Lantian1 (134), Lantian2 (135), Wangshunshan (136)
Danfeng		P	Green		Wine^§^	1	Danfeng2 (137)
			Red		Wine^§^	1	Danfeng1 (138)
Yunxi		P	Red		Wine^§^	3	Yunxi1 (139), Yunxi2 (140), Yunxi3 (141)
Duan		P	Red		Wine^§^	3	Douan1 (142), Douan2 (143), Douan3 (144)
Zhijiang		D	Red		Wine^§^	3	Gaoshan-1 (145), Gaoshan-2 (146), Shuijing Brier (147)
Hongjiang		D	Red		Wine^§^	2	Xuefengshan1 (148), Xuefengshan2 (149)
Chongyi		D	Green		Wine^§^	1	Baiyu (150)
			Red		Wine^§^	4	Chongyi1 (151), Chongyi2 (152), Chongyi3 (153), Junzi (154)
Yushan		D	Red		Wine^§^	1	Tangwei (155)
Tonghua		LV	Green		Wine	1	Vidal Blanc (156)
		AM	Red		Wine	5	Shuanghong (157), Shuangyou (158), Tonghua1 (159), Zuoshan1 (160), Zuoshan2 (161)
		VAM	Red		Wine	4	Beichun (162), Beihong (163), Gongniang1 (164), Gongniang2 (165)

^a^ V, *V. vinifera*; LV, *V. labrusca* × *V. vinifera*; AL, *V. aestivalis* × *V. labrusca*; R, *V. rotundifolia*; L, *V. labrusca*; VR, *V. vinifera* × *V. riparia*; LR, *V. labrusca*. × *V. riparia*; P, *Vitis pentagona*; D, *V. davidii*; AM, *V. amurensis*; VAM, *V.vinifera* × *V. amurensis*; ^b^ Rs, Rootstock; ^c^ Number in parentheses following the cultivar indicates the serial number; ^§^ These cultivars are already used as wine grapes or show a great potential for making wine.

The contents of *trans*-resveratrol in the analyzed *V. vinifera* table grapes from Yangling and Yantai were in the range of 671.2 to 841.8 mg kg^−1^ of cane FW with a median of 782.4 mg kg^−1^ of cane FW; and 434.5 to 980.9 mg kg^−1^ of cane FW with a median of 633.4 mg kg^−1^ of cane FW, respectively. For the *V. vinifera* table grapes from the Yangling Grape Germplasm Repository the highest value was found in cv. ‘Shandongzaohong’ (841.8 mg kg^−1^ of cane FW), while the lowest one found in cv. ‘Queen of the Vineyard’ (671.2 mg kg^−1^ of cane FW). Three cultivars from Yantai containing higher levels of *trans*-resveratrol were ‘Fenghuang 51’ (980.9 mg kg^−1^ of cane FW), ‘Autumn Royal’ (952.4 mg kg^−1^ of cane FW), and ‘Muscat Mathiasz Janosne’ (946.9 mg kg^−1^ of cane FW). The *trans*-resveratrol content in ‘Victoria Blanc’ from Yantai was found to have the lowest value of 434.5 mg kg^−1^ of cane FW.

Compared with other grape genotypic groups, table grape cultivars of *V. labrusca* and *V. vinifera* hybrids in both grape repositories in this study had much lower *trans*-resveratrol contents. The contents of *trans*-resveratrol in table grapes ranged from 396.8 to 722.9 mg kg^−1^ of cane FW with a median of 564.2 mg kg^−1^ of cane FW in Yangling and from 320.6 to 549.3 mg kg^−1^ of cane FW with a median of 450.8 mg kg^−1^ of cane FW in Yantai. The cultivars ‘Ikawa 1025’ from Yangling and ‘Takasumi’ from Yantai had the highest amounts of *trans*-resveratrol among table grapes, and their concentrations were 722.9 and 549.3 mg kg^−1^ of cane FW, respectively. The lowest values were in the cultivar of ‘Golden Queen’ (396.8 mg kg^−1^ of cane FW) from Yangling and ‘Triumph’ (320.6 mg kg^−1^ of cane FW) from Yantai.

Only the table grape cultivar ‘Conquistador’, a hybrid between *V. aestivalis* and *V. labrusca* from the Yangling Grape Germplasm Repository, had a similar *trans*-resveratrol amount to the median of the table grapes of *V. vinifera* from the same repository. ‘Alachua’, the other table grape cultivar of *V. rotundifolia* from Yangling, had a relatively low value of 416.8 mg kg^−1^ of cane FW. The *trans*-resveratrol contents were 576.5 and 467.4 mg kg^−1^ of cane FW for ‘Moore's Diamond’ and ‘Concord’, two juice cultivars of *V. labrusca* from Yantai, respectively. ‘Bacco Noir’, a wine grape from Yantai, a *V. vinifera* and *V. riparia* hybrid, posted a value of 694.3 mg kg^−1^ of cane FW. A relatively high amount of *trans*-resveratrol was found in the only rootstock cultivar ‘Beta’ in Yantai, a hybrid between *V. labrusca* and *V. riparia*, reaching 773.5 mg kg^−1^ of cane FW.

The average *trans*-resveratrol contents in grape canes of the two grape germplasm collections are listed in Table 2, based on the classifications by different genotypes (*V. vinifera*, *V. labrusca*, or *V. labrusca* and *V. vinifera* hybrids), different fruit traits (red, including all rouge, purple, and noir cultivars; or green, including all yellow cultivars), and different uses (table, wine, or juice grapes). Further statistical analysis showed that there were significant differences in the amounts of *trans*-resveratrol in grape canes within and among different groups and similar variations in the corresponding groups from the two grape germplasm collections (Table 2).

For all grape cultivars of three species in this study the average *trans*-resveratrol content in grapes of *V. vinifera* was 937.9 mg kg^−1^ of cane FW, and significantly higher than that (571.3 mg kg^−1^ of cane FW) of the *V. labrusca* and *V. vinifera* hybrids in Yangling (*P* < 0.05 and *P* < 0.01). The cultivars of *V. vinifera* from Yantai had significantly higher amounts of *trans*-resveratrol than did both *V. labrusca* and *V. labrusca* and *V. vinifera* hybrids (*P* < 0.05 and *P* < 0.01). However, there were no significant differences between cultivars of *V. labrusca* and those of *V. labrusca* and *V. vinifera* hybrids in Yantai. With regard to grape cultivars’ purpose of uses both originating from *V. vinifera* and *V. labrusca* and *V. vinifera* hybrids, wine grapes had significantly higher *trans*-resveratrol than did table grapes in both Yangling and Yantai (*P* < 0.05 and *P* < 0.01), whereas there were no significant differences between juice cultivars of *V. labrusca* and table cultivars of *V. vinifera* and *V. labrusca* and *V. vinifera* hybrids in Yantai. In addition, no significant differences of *trans*-resveratrol contents were observed between the red and green cultivars among different species in both Yangling and Yantai (Table 2).

The results of the study on the two grape germplasm repositories indicated that the amounts of *trans*-resveratrol in one-year-old canes of various cultivars were strongly influenced by their genetic background. *trans*-Resveratrol synthesis in plants can usually be triggered by exogenous stress factors, such as UV irradiation and the presence of pathogenic fungi, as discussed previously, but its synthesis in grape canes during the dormant season with low fungal infection conditions and weak UV irradiation might be a non-specific response to those stresses. Therefore, *trans*-resveratrol can be considered as a normal constituent of grape canes, as the similar results reported by Langcake and Pryce [32] in the lignified stems of cv. Müller-Thurgau.

**Table 2:** Comparison of *trans*-resveratrol contents (mean value ± S.D., mg kg^−1^ of FW) in grape cane among different genotypic groups or purpose of uses of two grape germplasm repositories.

Genotype group or purpose of use	Yangling	Yantai
All cultivars in this study	816.0 ± 252.5	706.2 ± 191.2
All cultivars of V^a^	937.9 ± 210.0***^T^	754.9Aa ± 173.4
All cultivars of LV^b^	571.3 ± 103.8	453.1Bb ± 62.5
All cultivars of L^c^	‒^d^	521.9Bb ± 77.1
All wine grapes of V & LV	950.8 ± 213.7***^T^	796.3Aa ± 169.7
All table grapes of V & LV	610.2 ± 135.9	618.9Bb ± 176.7
All juice grape of L	‒^d^	521.9Bb ± 77.1
All red cultivars of V & LV	843.8 ± 289.7	733.4 ± 194.6
All green cultivars of V & LV	796.9 ± 174.2	668..8 ± 187.9
All wine grapes of V	950.7 ± 213.7*^T^	796.3 ± 169.7*^T^
All table grapes of V	804.6 ± 116.4	689.9 ± 160.8

The lowercases and uppercases mean significant variation of average *trans*-resveratrol in grape cane of Yantai Grape Germplasm Repository among different genotypic groups or purpose of uses at *P* < 0.05 and *P* < 0.01 levels (ANOVA), respectively; and different letters indicate significant variation; ^a^ V, *V. vinifera*; ^b^ LV, hybrid of *V. labrusca* and *V. vinifera*; ^c^ L, *V. labrusca*; ^d^ Not available; ^T^ * and *** indicate significant difference of average *trans*-resveratrol in grape cane in the same grape germplasm repository between different genotypic groups or purpose of uses at *P* < 0.05 and *P* < 0.01 levels (Student’s *t*-test), respectively.

### 2.2. Occurrence and prediction of trans-resveratrol in main grape cultivars from China and comparison with other known sources

The concentration ranges and averages with standard deviations of *trans*-resveratrol in the main cultivars from three major grape production areas and four important distribution centers of Chinese wild *Vitis* species are shown in Table 3. Occurrences of *trans*-resveratrol in the main known sources and transgenic plants were summarized in Table 4.

**Table 3:** Occurrence and potential value estimation of *trans*-resveratrol in main grape cultivars from seven major grape production regions.

Major region	Usage	Main grape cultivars^a^	*trans*-Resveratrol occurrence^b^	Area^c^	Predicted yield^d^	Estimated economic output^e^
C1	Table/ raisin	1, 2, 3, 4, 5, 6, 7.	774.0BCbc ± 121.8 (589.9–983.9)	96.2	74.5 (56.7–94.7)	149.0–223.5
C2	Wine	74, 75, 80, 81, 83, 84, 85, 86, 87, 88, 89, 90, 91, 92, 93, 104,105.	834.1De ± 202.4 (608.9–1452.9)	48	40.0 (29.2–69.7)	80.0–120.0
	Table	67, 70, 71, 72, 73, 107, 108, 110, 111, 112, 113, 114, 116, 117, 118, 119, 121, 122, 123, 124, 126, 127.	610.1Aa ± 167.1 (320.6–980.9)	100.3	61.2 (32.2–98.4)	122.4–183.6
C3	Wine	8, 9, 10, 11, 12, 13, 14, 15, 27, 28, 31, 34, 44, 45, 47.	937.8CDcd ± 175.7 (763.8–1369.6)	20	18.8 (15.3–27.4)	37.6–56.4
	Table	16, 17, 49, 50, 51, 52, 53, 57, 58, 59, 61, 64.	613.3Aa ± 155.4 (396.3–881.9)	15.9	9.8 (6.3–14.0)	19.6–29.4
W1	Wine	157, 158, 159, 160, 161, 162, 163, 164, 165.	889.7CDde ± 62.2 (818.7–964.6)	40	35.6 (32.7–38.6)	71.2–106.8
W2	Wine^§^	134, 135, 136, 138, 139, 140, 141.	700.6ABab ± 64.9 (564.6–767.8)	4.8	3.4 (2.7–3.7)	6.8–10.2
W3	Wine^§^	145, 146, 147, 148, 150, 151, 152, 154, 155.	1048.9Ef ± 137.9 (889.8–1285.9)	19.3	20.2 (17.2–24.8)	40.4–60.6
W4	Wine^§^	142, 143, 144.	838.8CDcd ± 30.9 (823.5–850.4)	10	8.4 (8.2–8.5)	16.8–25.2
Sum.^f^				354.5	271.9 (200.5–379.8)	543.8–815.7

The lowercases and uppercases mean significant variation at 0.05 and 0.01 level, respectively; and the values in any two groups with different letters indicate significant variation (P < 0.05 or P < 0.01); ^a^ The numbers in the following blanks represent the same accession numbers to Tab. 1; ^b^ Mean values ± S.D., range values in parenthesis, all values in mg kg^−1^ of cane FW; ^c^ Data of cultivated area of each major region (MOA, 2006), all values in kha; ^d^ Mean values ± S.D., range values in parenthesis, all values in ton of *trans*-resveratrol year^−1^; ^e^ Estimated economic output range values of each major region, all values in US $ million year^−1^; ^f^ Summation, range values in parenthesis; ^§^ These cultivars are already used as wine grapes or show a great potential for making wine.

**Table 4:** Occurrence of *trans*-resveratrol in the main known sources.

Sources		Cultivars or types		*trans*-Resveratrol		Ref.	
*Polygonum cuspidatum*		R. japonica		64^d^		[10]	
		R. × bohemica		23^d^			
		R. sachalinensis		29^d^			
		HZ, MB		3770^d^, 2960^d^		[37]	
Grape juice		Palomino fino		2.4^l^		[5]	
Grape berry		Muscadine		5.2-26.4^f^		[42]	
Grape seed		Gamay		3.9^f^		[43]	
		Pinot Noir		588^d^		[23]	
Grape skin		Pinot Noir		118^d^		[23]	
		Gamay		6.8^f^		[43]	
		Palomino fino		15.7^f^		[5]	
Grape pomace		Palomino fino		192^d^		[21]	
		Muscadine		22.1-84.2^d^		[42]	
Red wine		Muscadine		0.4-2.0^l^		[42]	
		Other red wine^a^		0.2-14.3^l^		[30]	
White wine		White wines^b^		≈ 0.1^l^		[31]	
Peanut		Jinpoong		1.3^f^		[3]	
		NC-7, Çom, Gazipaşa, Florispan , Çerezlik 5025, Çerezlik PI-355276		0.03-1.92^d^		[44]	
		Other peanut cultivars^c^		0.02-1.79^d^		[6]	
Peanut root		Jinpoong		1.19^f^		[3]	
		Tainan 9, Tainan 11, Tainan 12		15-1330^d^		[29]	
Strawberry		Allstar		0.09^f^, 0.83^d^		[8]	
Blueberry		Highbush Michigan		0.03^f^, 0.02^d^		[7]	
		Lowbush "wild" Nova Scotia		0.01^f^, 0.02^d^			
Bilberry		Polish		0.02^f^, 0.02^d^		[7]	
Pistachio		Ohadi, Uzun, Kırmızı, Halebi, Siirt		0.09-1.67^d^		[44]	
Chocolate		Dark color		2.0^f^		[45]	
Hop		Hop cultivars^e^		0.10-2.28^d^		[9]	
Hop^ tg^		Tettnang		13^d^, 2.7^f^		[46]	
Lettuce^tg^		Unknown		56.4^f^		[28]	
Tobacco^tg^		W38		9.3^f^		[47]	
Wheat^tg^		Florida, Combi		35-190^f^		[48]	
Oilseed rape^tg^		Drakkar		361^f^		[49]	
Kiwifruit^tg^		Hayward		182^f^		[50]	

^a^ Including Cabernet Sauvignon (*n* = 40), Tempranillo (*n* = 12), Cabernet Franc (*n* = 8), Liatiko (*n* = 4), Xinomauro (*n* = 10), Muscat Bailey A (*n* = 5), Zinfandel (*n* = 5), Agiorgitiko (*n* = 9), Marzemino (*n* = 3), Blaufränkisch (*n* = 18), Portugieser (*n* = 9), Zweigelt (*n* = 10), Negroamaro (*n* = 3), Teroldego (*n* = 3), Nero d’Avola (*n* = 5); ^b^ 21 French wines, 23 American wines, 18 from Hungary and Central Europe, 20 from Italy, 7 from Canada, 2 from South America, and 8 from Australia; ^c^ Including Spanette, Pearl, NC-18016, Early Bunch, White’s Runner, Florunner C1, GA 207-3-4, GA 207-2, NC-9, Florispan C1, NC-17291, Dixie Runner, PI-337396-FAV70, Florispan C3, Small White Spanish; ^e^ Including Hersbrucker Spat, Spalter, Saphir, Hallertau Mittlefrüher, Smaragd, Hallertau Tradition, Wye Target, Nugget, Hallertau Magnum, Hallertau Taurus, Saaz, Sladeck, Premiant, Willamette, Cascade, Tomahawk, Simcoe, Warrior; ^d^ mg kg^-1^ dry weight basis; ^f^ mg kg^-1^ fresh weight basis.^l^ mg L^−1^; ^tg^ Transgenic plants.

The observed *trans*-resveratrol contents in grape canes were higher than the reported amounts in most of the other known sources, with a few exceptions (Table 3, Table 4). Furthermore, *trans*-resveratrol occurs in *Polygonum cuspidatum* and peanut roots in the range from a few tens to a few thousands mg kg^−1^ dry weight, showing a marked fluctuations appearing to be related to various factors [10,37,29]. In contrast, grape canes represent a novel and stable source of *trans*-resveratrol.

In this study, 165 grape cane samples were used to estimate the annual yields and the economic outputs of *trans*-resveratrol from grape cane wastes of seven major grape production regions of China (Table 3). Predicted annual yields of *trans*-resveratrol from each region were calculated with the mean and range values shown in Table 3, with an approximate annual grape cane production rate of 1 t ha^−1^ [38] and cultivated area of each major production region [39]. Estimated annually economic outputs of each region were generated from the average yields values of *trans*-resveratrol, at a commercial price of food-grade product ranging from US$ 2,000 to US$ 3,000 kg^−1^ reported by Baur and Sinclair when they evaluated the costs of daily or yearly *trans*-resveratrol intake for a human [40]. The total value of *trans*-resveratrol from grape cane wastes in China could reach up to US$ 543.8–815.7 million year^−1^ based on cultivation data of 2006. Actually, the vineyard area in China, with a steady increase year after year, reached approximately 475 mha in 2009 [41]. Recovery and utilization of *trans*-resveratrol from these agricultural pruning wastes, as a sideline production, indicates a huge economic potential.

## 3. Experimental

### 3.1. Plant material

One hundred and sixty five (165) grape cane samples used in this study (Table 1), including seven raisin grapes of *V. vinifera* from Turpan, Xinjiang (C1), eight wine grapes of *V. vinifera* from Helan, Ningxia (C2), 51 grape cultivars from the experimental vineyard of grape germplasm repository of College of Enology, Northwest A&F University at Yangling, Shaanxi (C2), 67 grape cultivars from the grape germplasm collection of Changyu Pioneer Wine Co. Ltd in Yantai, Shandong Province (C3), one wine grape hybrid of *V. labrusca* and *V. vinifera*, five wine grapes of *V. amurensis*, and four wine grape hybrids of *V. vinifera* and *V. amurensis* from Tonghua, Jilin (W1), 11 genotypes of *V. pentagona* from Lantian, Shaanxi, Danfeng, Shaanxi, and Yunxi, Hubei, and Douan, Guangxi (W2, W4), 11 grape cultivars or genotypes of *V. davidii* from Zhijiang, Hunan, Hongjiang, Huan, Chongyi, Jiangxi, and Yushan, Jiangxi (W3). The ideal one-year-old canes with moderate growth vigor (approximately 0.8–1.0 cm diameter) were collected from the above locations during the 2008 pruning practice. All cane samples were frozen in liquid nitrogen, ground through a 0.5 mm sieve using an electrical grinder (final particle size < 0.5 mm), stored under vacuum in labeled plastic containers, and then stored at −20 °C in a freezer until extraction.

### 3.2. Chemicals

*trans*-Resveratrol standard (purity >97%) was purchased from Sigma-Aldrich Chemical Co. (Shanghai, China). Methanol and acetonitrile were HPLC grade solvents from Tianjin Kermel Chemical Reagent Co. Ltd. (Tianjin, China). Analytical grade acetic acid was from Xi’an Chemistry Factory (Xi’an, China). Water was purified using the Milli-Q system (Millipore, Bedford, MA, USA).

### 3.3. Extraction of trans-resveratrol from grape canes

Triplicate samples of ground grape cane (5 g, fresh weight) were extracted three times with acidified methanol solution (40 mL, 1 N HCl/methanol/water, 1/80/19, v/v/v), and extraction was performed under continuous stirring (600 rpm) at 20 °C for 1h in an external water bath. The extracts were centrifuged at 8,000 g for 15 min at 4 °C using a Sorvall RC-5C Plus centrifuge (Kendro Laboratory Products, Newton, CT, USA). All the supernatants were combined in a 250 mL flask and concentrated in a Büchi RE-111 evaporator (Switzerland) at 35 °C to a volume of 10 mL. The final concentrate solution was filtered through a 0.22 µm nylon micro-membrane and stored at −40 °C until analysis.

### 3.4. Chromatographic analysis

The chromatographic analyses were carried out on a Shimadzu liquid chromatograph system (Shimadzu Corp, Kyoto, Japan) equipped with a quaternary pump coupled with a photodiode array detector and a UV-Vis detector.

**Figure 4:**
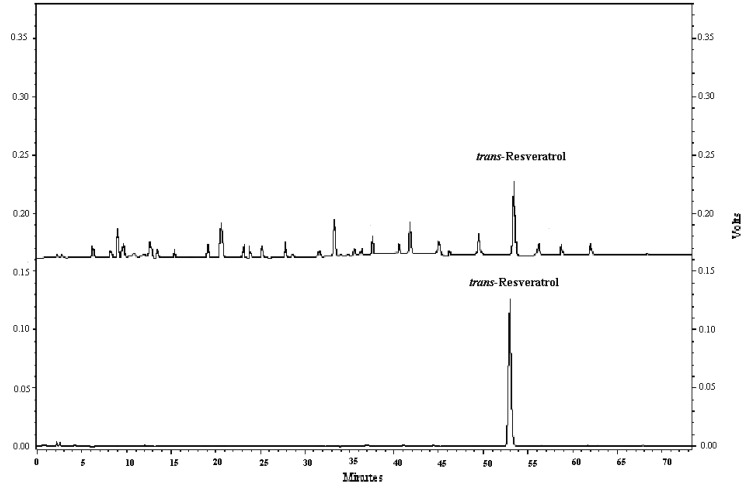
Typical HPLC chromatographies of grape cane extracts (top) and *trans*-resveratrol standard (bottom) captured at 306 nm.

Samples were injected onto a Shim-Pack VP-ODS C_18_ column (250 mm × 4.6 mm, 5 μm) at room temperature. The mobile phase was acidified water containing 3% acetic acid (A) and acetonitrile (B). The gradient program is as follows: B: 0.00–5.00 min, 0–8.5%; 5.00–16.50 min, 8.5–2.0%; 16.50–35.00 min, 2.0–18%; 35.00–50.00 min, 18–20%; 50.00–65.00 min, 20–30%; 65.00–70.00 min, 30–0%. The column held at 30 °C was flushed at a flow rate of 0.8 mL/min. The DAD detector was applied to ascertain the maximum absorbance wavelength of *trans*-resveratrol within a range of 200 to 400 nm. The UV-vis detector was conducted at 306 nm for quantitation of *trans*-resveratrol in grape cane extracts with the external standard. Chromatographic identification and confirmation of *trans*-resveratrol were based on comparing retention times with the authentic standard and on-line UV absorption spectrum data. Results were acquired and processed by the Shimadzu Workstation CLASS-VP 6.12 software (Shimadzu Corp, Kyoto, Japan). Typical HPLC chromatographies of grape cane extracts and *trans*-resveratrol standard are shown in Figure 4.

### 3.5. Statistical analysis

Mean values of each grape cane sample were from three replicates, and were employed for further analysis. For the data of two grape germplasm repositories, the cultivar variations of *trans*-resveratrol contents in one-year-old canes were evaluated at the germplasm level, and the box-and-whisker plots, which are helpful in interpreting the distribution of data, were generated by the software of Sigmaplot version 10.0 (Systat Software Inc., US) to display range, median, and distribution density of variables in sample size. The Student’s independent-sample *t-*test and one-way analysis of variance (ANOVA) were carried out by SPSS version 10.0 (SPSS, Inc., Chicago, IL, USA)

## 4. Conclusions

Grape canes are generated in a substantial quantity as agricultural pruning wastes in the grape industry. *trans*-Resveratrol has been proven to be a normal constituent of grape canes and its content varies largely with different genotypic groups as well as their purpose of uses. Compared with other known sources, the *trans*-resveratrol content in grape cane wastes is high enough to indicate they are a potentially new and stable commercial source. Occurrence of *trans*-resveratrol in main grape cultivars cultivated in seven major grape production regions in China shows a great potential economic value.
